# Avian Influenza Vaccination of Poultry and Passive Case Reporting, Egypt

**DOI:** 10.3201/eid1812.120616

**Published:** 2012-12

**Authors:** Timothée Vergne, Vladimir Grosbois, Yilma Jobre, Ahmed Saad, Amira Abd El Nabi, Shereen Galal, Mohamed Kalifa, Soheir Abd El Kader, Gwenaëlle Dauphin, François Roger, Juan Lubroth, Marisa Peyre

**Affiliations:** Author affiliations: Centre International de Recherche en Agriculture pour le Développement, Montpellier, France (T. Vergne, V. Grosbois, F. Roger, M. Peyre);; French Agency for Food, Environmental and Occupational Health and Safety, Maisons-Alfort, France (T. Vergne);; Food and Agriculture Organization of the United Nations, Cairo, Egypt (Y. Jobre, A. Saad, A. Abd El Nabi);; Central Laboratory for Veterinary Quality Control of Poultry Production, Giza, Egypt (S. Galal, M, Kalifa);; General Organisation for Veterinary Services, Cairo (S. Abd El Kader);; Food and Agriculture Organization of the United Nations, Rome, Italy (G. Dauphin, J. Lubroth)

**Keywords:** Avian influenza, vaccination, epidemiology, capture-recapture, underreporting, surveillance, Egypt, influenza, zoonoses, poultry, virus

## Abstract

We investigated the influence of a mass poultry vaccination campaign on passive surveillance of highly pathogenic avian influenza subtype (H5N1) outbreaks among poultry in Egypt. Passive reporting dropped during the campaign, although probability of infection remained unchanged. Future poultry vaccination campaigns should consider this negative impact on reporting for adapting surveillance strategies.

Egypt reported its first occurrence of highly pathogenic avian influenza (HPAI) virus subtype H5N1 in poultry on February 16, 2006 (*1*), and its first case in a human on March 20, 2006. As of June 2011, Egypt was the country most affected by HPAI (H5N1) outside of Asia (*2*). Vaccination of domestic (backyard) and commercial poultry, which began in March 2006, and other measures were implemented to control the disease, but outbreaks among poultry and humans continued to be regularly reported from various districts located mainly in the delta region of the country (*3*). In July 2009, vaccination of domestic poultry was stopped (*4*). The objective of this study was to assess the effect of vaccination of domestic poultry on the passive reporting of HPAI (H5N1) cases among poultry. The completeness of the passive surveillance of poultry cases at the district level during and after the mass vaccination campaign was estimated by using a 4-source capture-recapture method (*5*).

## The Study

Two periods were selected for study: period 1 (December 2008–June 2009), during which mass vaccination of backyard poultry was ongoing, and period 2 (December 2009–June 2010), during which mass vaccination had ceased (Figure). Vaccination of commercial poultry in Egypt against avian influenza (AI) continued throughout the study period. In Egypt, the district level is the smallest administrative unit used for defining surveillance and control strategies related to HPAI (H5N1) among poultry. Thus, we used the district level to estimate the occurrence of HPAI (H5N1) during the 2 study periods.

**Figure:**
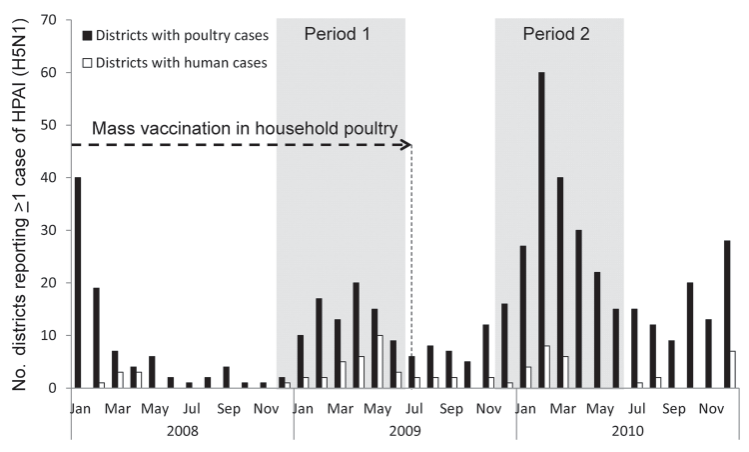
Number of districts in which avian influenza (H5N1) virus infection was detected among poultry and humans during and after a campaign of mass vaccination of backyard poultry, Egypt, January 2008–December 2010. Activity was identified by active, passive, or participatory surveillance at the district level. Cessation of the vaccination campaign appeared to cause a large increase in the number of infected districts that were detected during Period 2. Shading indicates periods of study. HPAI, highly pathogenic avian influenza.

HPAI (H5N1) circulation in poultry is recorded by the national surveillance of poultry coordinated by the General Organization for Veterinary Services (GOVS) in conjunction with the Central Laboratory for Quality Control of Poultry Production, based in Cairo. During the 2 study periods, poultry surveillance was structured into 3 distinct protocols: 1) passive surveillance reliant on disease reporting by farmers; 2) active surveillance of preslaughter poultry and high-risk areas (areas defined by GOVS as high risk based on specific criteria, such as the density of the poultry population and the detection of HPAI (H5N1) cases among humans and poultry in previous months); and 3) the community based animal health outreach program, a participatory surveillance network that relies on traditional information networks to track down and confirm HPAI (H5N1) events in areas where virus circulation is suspected.

The lists of cases collected through these complementary protocols by GOVS and the Central Laboratory for Quality Control of Poultry Production was provided by the Emergency Centre for Transboundary Animal Diseases Unit of the Food and Agriculture Organization in Egypt. Since 2006, the government has required the culling of poultry flocks in which cases of avian influenza were detected. As part of the initial program, government compensation for culled birds was also implemented, but because of misuse, the compensation program was stopped in 2007.

It is assumed that most human cases of influenza (H5N1) are linked to infections in poultry (*3*,*6*,*7*); thus, we postulated that a human case within a district revealed avian influenza (H5N1) virus circulation among poultry within that district. For methodological purposes, we hypothesized that a human could not get the infection from outside the district of residence. In Egypt, the protocol for surveillance of human influenza cases is based on reporting of suspected cases in district hospitals followed by confirmation of infection by the Central Public Health Laboratory in Cairo and the US Naval Medical Research Unit 3 (*8*). Data for human cases were obtained from the World Health Organization (www.who.int/csr/don/archive/country/egy/en/). From this list of cases, 1 case from period 2 was excluded because exposure to sick or dead poultry was not confirmed.

Capture-recapture methods were introduced in the field of ecology for estimating the size of wild populations and subsequently adapted to surveillance of infectious diseases in humans and animals (*5*,*9*,*10*). After accounting for the small sample sizes in our study (*11*), we used log-linear models to model cross-detection frequency data (Table 1) (*12*). The best model was selected by using the Akaike information criterion and projected onto the “no detection” history to estimate the frequency of districts where the virus was circulating but not reported (*5*). Details about our method can be found in the Technical Appendix. 

**Table 1:** Avian influenza vaccination and disease detection history of districts reporting highly pathogenic avian influenza (H5N1) in poultry and humans, Egypt*

Detection history					No. districts reporting infection	
AS†	PS‡	Part S§	SH¶		Period 1	Period 2
1	0	0	0		13	6
0	1	0	0		22	38
0	0	1	0		4	5
0	0	0	1		15	4
1	1	0	0		3	7
1	0	1	0		1	1
1	0	0	1		7	1
0	1	1	0		1	20
0	1	0	1		2	3
0	0	1	1		1	1
1	1	1	0		1	2
1	1	0	1		2	1
1	0	1	1		0	0
0	1	1	1		0	5
1	1	1	1		1	2
0	0	0	0		NK#	NK

*Columns1–4 describe detection of infection by various protocols: 1 = infection reported using protocol; 0 = no infection reported using protocol, e.g., the first line corresponds to the districts in which infection was detected only by the active surveillance of poultry. Period 1, Dec 2008–Jun 2009; period 2, Dec 2009–Jun 2010. †Active surveillance of poultry. ‡Passive surveillance of poultry. §Participatory surveillance of poultry. ¶Surveillance in humans. #NK, not known.

For period 1, the best model was the independent model that assumed no interaction between any of the 4 detection sources. For period 2, a significant positive interaction (p<0.05) was detected between passive surveillance and participatory surveillance. The extent of the 2 outbreaks appeared similar at district level. The actual number of districts that reported infections in poultry was estimated to be ≈126 and 133, respectively, for period 1 and period 2 (Table 2). As a consequence, the Figure suggests that surveillance was affected heavily by underreporting during period 1. The completeness of poultry surveillance at the district level increased, rising from 46% to 69% between the 2 periods. Moreover, the sensitivity of the passive surveillance among poultry during period 2 was estimated to be more than twice as high as during period 1.

**Table 2:** Estimated surveillance parameters for avian influenza virus infection in Egypt*

Estimated parameters	Period 1			Period 2	
	Point estimates	95% CI		Point estimates	95% CI
Number of districts with infection among poultry	126	107–159		133	118–160
Completeness of the 4 detection sources	0.58	0.46–0.68		0.72	0.60–0.81
Completeness of surveillance among poultry	0.46	0.36–0.54		0.69	0.58–0.78
Completeness of passive surveillance	0.25	0.20–0.30		0.59	0.49–0.66

*Period 1, Dec 2008–Jun 2009; period 2, Dec 2009–Jun 2010.

During period 2, participatory surveillance targeted zones where HPAI (H5N1) virus circulation had been informally reported in poultry, potentially overlapping spontaneous reports and resulting in a direct positive dependence on passive surveillance. No positive dependence between surveillance of humans and active or participatory surveillance of poultry was detected, even though such dependence could have been predicted because of possible investigations into poultry cases after human cases were reported.

The assumption of no indirect dependence between the 4 detection sources is the most critical aspect of this study. It is possible that all 4 sources had a higher probability of detecting districts with a high incidence of disease than a low incidence of disease. As a consequence, for each period, this positive indirect dependence is likely to result in underestimation of the true number of districts where HPAI (H5N1) virus was circulating in poultry (*5*), causing some districts that had few outbreaks to go uncounted. The consequence of such an underestimation is a slight overestimation of the completeness of each detection source. However, because this potential bias occurred for both periods, it should not influence the overall trend estimated between the 2 periods.

## Conclusions

Our findings support the hypothesis that mass avian influenza vaccination of domestic poultry may negatively affect passive surveillance for influenza infection among poultry (*4*). This phenomenon could be caused by changes in the clinical features of the disease linked to vaccination (e.g., lower mortality rate, fewer clinical signs). However, these changes should be considered negligible because of the limited efficacy of this vaccination strategy in the field (*4*). A disproportionate trust in the benefits of AI vaccination might be a more plausible explanation. This trust could lead to failure of the community to recognize the disease, based on the assumption that poultry vaccinated against HPAI (H5N1) could no longer be infected.

This study supports an updated interpretation of the evaluation of the HPAI (H5N1) control program in Egypt. We conclude that if reported outbreaks are the only data considered, the effectiveness of AI vaccination will be overestimated. Our findings stress the critical importance of the quality of data used in the evaluation of animal and public health control programs and the necessity to evaluate reporting rates.

Technical Appendix Description of multi-source capture-recapture method using log-linear models.
