# Supplementary material for: Avian Influenza Vaccination of Poultry and Passive Case Reporting, Egypt
Source: Emerg Infect Dis. 2012 Dec;18(12):2076–8. doi: 10.3201/eid1812.120616 (PMC3557869; doi:10.3201/eid1812.120616)
Supplement: Technical Appendix — Description of multi-source capture-recapture method using log-linear models. [file 12-0616-Techapp-s1.pdf]

Article DOI: http://dx.doi.org/10.3201/eid1812.120616

Avian Influenza Vaccination of Poultry and Passive Case Reporting, Egypt

Technical Appendix

Multisource Capture-Recapture Method Using Log-Linear Models (*1*)

For the sake of clarity, we will consider the situation where each infected epidemiologic unit can be detected by 3 detection sources, which is the simplest multisource situation. Matching these 3 detection sources allows representing the data in a detection history dataset as presented in the Table. In principle, all combinations of detection can be observed from “detected by each of the 3 sources” (7th line in Table) to “detected by none of the sources” (8th line in Table). To each detection history is associated the corresponding number of infected epidemiologic units (4th column in Table). By construction, the observed frequency of infected epidemiologic units detected by none of the 3 sources (X_{000}) is missing and needs to be estimated for getting an unbiased estimation of the true number of infected epidemiologic units.

Table. Representation of a capture history dataset with 3 detection sources

Source 1	Source 2	Source 3	Frequency
1	0	0	X_{100}
0	1	0	X_{010}
0	0	1	X_{001}
1	1	0	X_{110}
1	0	1	X_{101}
0	1	1	X_{011}
1	1	1	X_{111}
0	0	0	X_{000}?

Log-linear models enable modeling the natural logarithm of observed frequencies of each capture histories as a linear combination of an intercept, detection sources main effects and, possibly, interaction terms. The presence of a structurally empty cell in the dataset prevents constructing a model that would take into account the highest-order interaction term (i.e. the 3-way interaction term in the case of 3 detection sources). The assumption of insignificance of the highest-order interaction is therefore a prerequisite for any application. For 3 detection sources,

the saturated model (taking into account all possible interactions between sources except the 3-way interaction) can be written as

$$
\begin{cases}
\ln(X_{111}) = \theta_0 + \lambda_1 + \lambda_2 + \lambda_3 + \lambda_{12} + \lambda_{13} + \lambda_{23} \\
\ln(X_{110}) = \theta_0 + \lambda_1 + \lambda_2 + \lambda_{12} \\
\ln(X_{101}) = \theta_0 + \lambda_1 + \lambda_3 + \lambda_{13} \\
\ln(X_{011}) = \theta_0 + \lambda_2 + \lambda_3 + \lambda_{23} \\
\ln(X_{100}) = \theta_0 + \lambda_1 \\
\ln(X_{010}) = \theta_0 + \lambda_2 \\
\ln(X_{001}) = \theta_0 + \lambda_3
\end{cases}
$$

with θ_0 being the intercept, λ_1, λ_2, λ_3 being the main effects of source 1, 2 and 3 respectively, λ_{12}, λ_{13} and λ_{23} being the interaction terms. Selecting the model presenting the best compromise between fit the data and parsimony (i.e., minimal complexity) is usually done using a backward stepwise procedure based on the Akaike information criterion.

Once the best model is selected, the estimation of θ_0 allows estimating the number of infected epidemiologic units that are not detected (X_{000}) with $\widehat{X_{000}} = \exp(\widehat{\theta_0})$ and therefore estimating the total number of infected epidemiologic units $\hat{N}$. The estimation of the completeness of a detection source can be obtained by dividing the number of infected epidemiologic units detected by that source by the estimated total number of infected epidemiologic units.

This 3 detection sources situation corresponds to the simplest multisource case, and extension to 4 detection sources or more can be performed using the same principle.

In multisource capture-recapture applications, if there are null frequencies for some detection histories, the estimation procedures may lead to extreme or even infinite estimates. In such circumstances, Hook and Regal (*1*) proposed a small sample adjustment of the data: with an odd (even) number of detection sources, they suggest to add the value of 1 to the observed frequencies of the detection histories corresponding to an even (odd) number of detection sources.

Underlying Assumptions

In epidemiology, capture-recapture applications generally assume that a number of underlying assumptions are met. These assumptions have to be assessed for any specific case study.

The first assumption is that the sources should be directly independent: being detected by 1 source should not change the probability of being detected by another source. A positive direct dependence occurs when this probability increases which ultimately results in an underestimation of the true infected population size. With log-linear modeling, this assumption can be relaxed by using interaction terms between the dependent sources (*1*).

The second assumption is that the sources should be indirectly independent: if different sources experience heterogeneity in the detection probabilities (some cases are more easily detected than others) the factors driving these heterogeneities for different sources should not be correlated. A positive indirect dependence occurs when the heterogeneity factors are positively correlated, which results again in an underestimation of the true infected population size. Indirect dependencies can most often be taken into account in log-linear models with the use of interaction terms between the dependent sources. Heterogeneities in detection probabilities that are uncorrelated among sources do not hamper the application of capture-recapture (*2*).

The third assumption is that there are no false-positives results. The presence of false-positive results artificially inflates the observed frequencies in the detection history dataset, and generally produces overestimations.

References

1. Hook EB, Regal RR. Capture-recapture methods in epidemiology: methods and limitations. Epidemiol Rev. 1995;17:243–64. PubMed

2. Hook EB, Regal RR. Effect of variation in probability of ascertainment by sources (“variable catchability”) upon “capture-recapture” estimates of prevalence. Am J Epidemiol. 1993;137:1148–66. PubMed
